# Detection of rock bridges by infrared thermal imaging and modeling

**DOI:** 10.1038/s41598-019-49336-1

**Published:** 2019-09-11

**Authors:** Antoine Guerin, Michel Jaboyedoff, Brian D. Collins, Marc-Henri Derron, Greg M. Stock, Battista Matasci, Martin Boesiger, Caroline Lefeuvre, Yury Y. Podladchikov

**Affiliations:** 1 Institute of Earth Sciences, University of Lausanne, Lausanne, 1015 Switzerland; 2 U.S. Geological Survey, Landslide Hazards Program, Menlo Park, California 94025 USA; 3 U.S. National Park Service, Yosemite National Park, El Portal, California 95318 USA

**Keywords:** Environmental sciences, Natural hazards

## Abstract

Characterization of rock discontinuities and rock bridges is required to define stability conditions of fractured rock masses in both natural and engineered environments. Although remote sensing methods for mapping discontinuities have improved in recent years, remote detection of intact rock bridges on cliff faces remains challenging, with their existence typically confirmed only after failure. In steep exfoliating cliffs, such as El Capitan in Yosemite Valley (California, USA), rockfalls mainly occur along cliff-parallel exfoliation joints, with rock bridges playing a key role in the stability of partially detached exfoliation sheets. We employed infrared thermal imaging (i.e., thermography) as a new means of detecting intact rock bridges prior to failure. An infrared thermal panorama of El Capitan revealed cold thermal signatures for the surfaces of two granitic exfoliation sheets, consistent with the expectation that air circulation cools the back of the partially detached sheets. However, we also noted small areas of warm thermal anomalies on these same sheets, even during periods of nocturnal rock cooling. Rock attachment via rock bridges is the likely cause for the warm anomalies in the thermal data. 2-D model simulations of the thermal behavior of one of  the monitored sheets reproduce the observed anomalies and explain the temperature differences detected in the rock bridge area. Based on combined thermal and ground-based lidar imaging, and using geometric and rock fracture mechanics analysis, we are able to quantify the stability of both sheets. Our analysis demonstrates that thermography can remotely detect intact rock bridges and thereby greatly improve rockfall hazard assessment.

## Introduction

Erosion processes in rock masses depend on properties of the intact rock and the characteristics of brittle structures^1^ such as joints and other discontinuities. In steep bedrock landscapes, rockfalls are a dominant process that modulate overall erosion rates^2–8^. In some terrains (particularly granitic), rockfalls are often manifest by detachments of rock flakes^9–14^ (exfoliation sheets) that form along surface-parallel fractures known as sheeting joints^15–18^. Exfoliation sheets are typically connected to the rock by so-called “rock bridges”, areas of intact (unfractured) rock where sheeting joints have yet to propagate. Sheeting joints develop close to the surface, usually at less than 30 m depth^17^ and are characterized by extensive fractures whose surface trace length (known also as persistence) can exceed 100 m^18,19^.

Sheeting joint persistence and rock bridge area both directly influence cliff stability^20–22^. For example, the presence of rock bridges over as little as just a few percent of the detachment surface can significantly increase the factor of safety by increasing apparent cohesion of a potentially unstable rock mass^23–26^. In addition, recent work^27,28^ has begun to explore the contribution of rock bridges to defining various failure modes for rockfalls and rock slides. These studies clearly indicate that the spatial distribution of rock bridges is critical to understanding the stability of jointed rock masses.

The importance of rock bridges and step-path geometries for slope stability analysis has been recognized for several decades^29–35^. The contribution of rock bridges has been implemented in numerical models of rock slope stability using apparent cohesion^36–38^ or areas of intact rock^24,39–43^. In addition, techniques for mapping rock bridges on images and/or textured 3-D point clouds have substantially improved in recent years^24,34,35,42,44,45^. Nevertheless, these post-rockfall mapping methods are subject to exposure biases^46,47^ which depend on the accessibility and/or visibility of the outcrop. For example, the spatial resolution of the image and/or 3-D point cloud, the completeness of the 3-D point cloud (presence of shadow areas), as well as the quality of color contrasts used to distinguish fresh and pre-existing weathered fractures limit the ability of observers to provide reliable rock bridge extent percentages. More fundamentally, these studies seek to identify former rock bridges, which are only exposed after rock detachment has occurred, whereas evaluating the contribution of rock bridges to rockfall hazard requires detection of rock bridges prior to detachment. Aware of these limitations, one recent study^26^ proposed to work directly before a rockfall event to map intact rock bridges by combining observations from remote sensing and field mapping investigations. Based on the Digital Rock Mass Rating^48^ (DRMR), these authors made recommendations to improve digital mapping of discontinuity traces and defined new factors to quantify the intact rock bridge trace intensity^49,50^ R_21_. However, their modified DRMR only represents rock bridges in the form of discontinuous traces that delimit “rock mass bridges” and, so far, remote detection of an actual intact rock bridge has not yet been performed.

To address this need, we employed InfraRed Thermography (IRT). IRT has been used successfully to detect loose rock sections (represented by cooler thermal anomalies) in the fields of civil engineering^51,52^ and mine safety^53–57^, with increasing applications in earth sciences^58–60^. In landslide science, IRT has been coupled with Terrestrial Laser Scanning (TLS) to obtain information on the degree of fracturing of a rock mass^61^. Compact rocks have a greater thermal inertia than fractured rocks, therefore fractured rocks should experience faster temperature variations. Consequently, by repeating IRT surveys during cooling and heating phases, it is possible to highlight thermal anomalies, which are strongly controlled by the degree of fracturing and weathering of the rock. This approach was applied to rock wall surfaces by several authors^59,62–65^ for mapping open fractures and specific features such as rock cavities, seepage, highly fractured zones and weathered zones. To optimize the thermal mapping of fractured areas for stability analysis, one study^66^ developed a MATLAB toolbox (THIMRAN). However, even though IRT is a fast, efficient and easily reproducible measurement technique, this study pointed out that IRT results provide only 2-D images, whereas a correct interpretation of thermal contrasts would require a 3-D model.

Due to the influence of direct sun radiation, daytime thermograms are affected by cold thermal anomalies in areas partially shaded from the sun. Cross-validation with 3-D data thus enables detection of anomalies generated solely by the surface geometry. Given these diurnal artifacts, the best IRT acquisitions are obtained during the night^59^. Other authors^67^ successfully classified natural elements along a rock slope (vegetation, debris, talus, intact rock, weathered and fractured rock) according to their thermal signature in early night. The thermal classification then helps to better delineate hazardous areas subject to rockfalls. By combining IRT with TLS and ground-based InSAR, one recent study^68^ characterized the stability of rock cliffs that are difficult or dangerous to access. The associated rockfall susceptibility map was based on extremely high surface temperature anomalies, structural analysis, and measurement of displacements supported by geomechanical analyses^68^. However, it should be noted that the influence of emissivity is rarely highlighted in most of these studies, even though it can be the main source of a difference in temperature between two rock types or between intact and oxidized surfaces^69,70^. An accurate estimate of the emissivity of the different studied surfaces is therefore needed to correctly interpret thermal contrasts.

Here we demonstrate the potential of IRT to remotely detect intact rock bridges. We report the results of infrared thermal monitoring, calibrated in temperatures and corrected for emissivity effect, performed over four hours on a 1000-m-tall cliff: the southeast face of El Capitan in Yosemite Valley (California, USA). We interpret the observation of warmer thermal anomalies at the surface of two well-delimited exfoliation sheets (Boot Flake and Texas Flake) as signs of the presence of rock bridges. This interpretation is supported by the results of 2-D thermal modeling that reproduces the Boot Flake’s thermal behavior. We derive an estimate of the size of the potential rock bridges by draping the IRT images on the TLS topographic data, which we then used to evaluate the stability conditions of both monitored exfoliation sheets.

## Study Site

**Figure 1:**
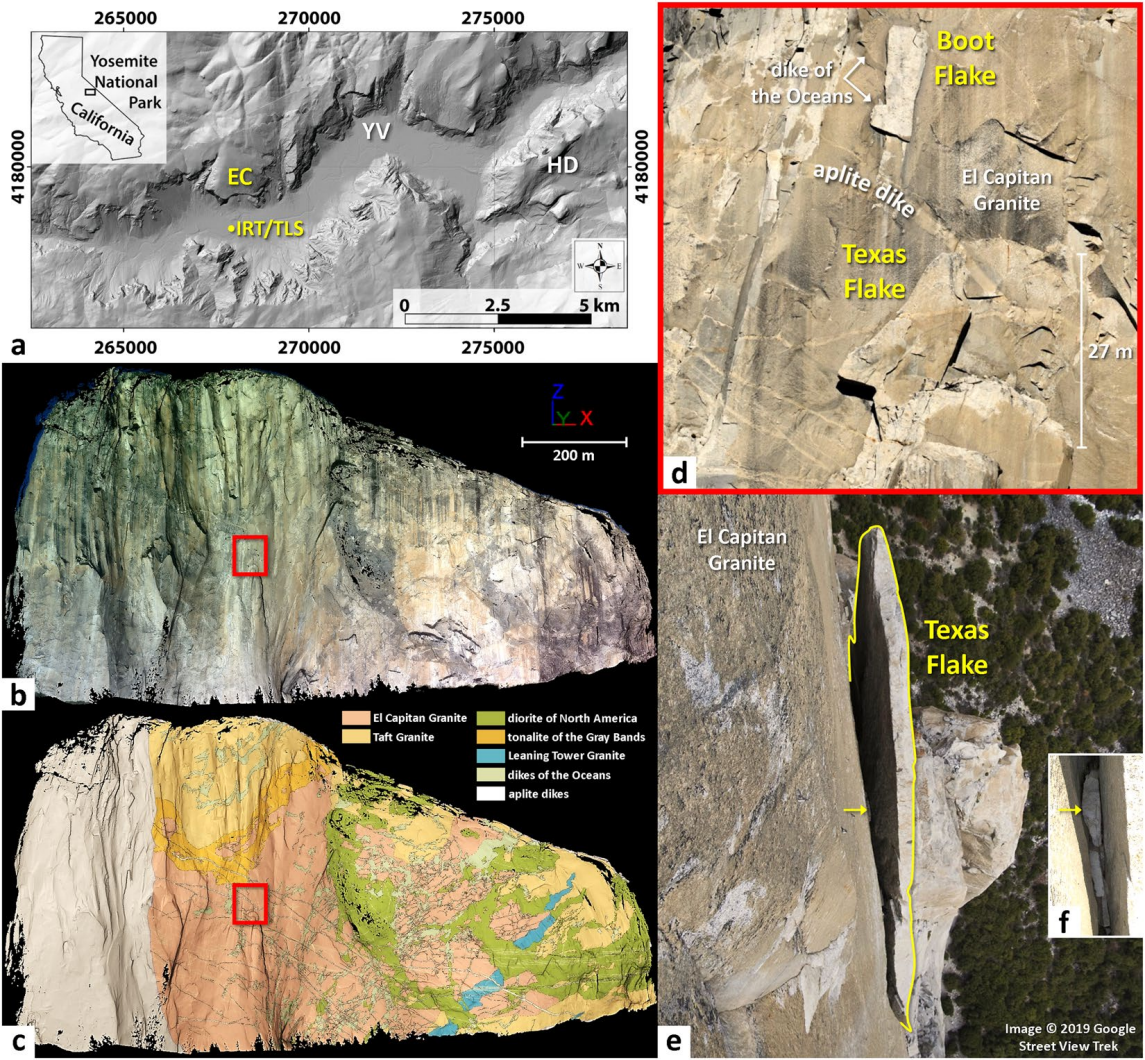
Location and geologic setting of the study area. (**a**) Aerial view of Yosemite Valley (California, USA). Key to abbreviations: EC = El Capitan; HD = Half Dome; YV = Yosemite Village. The yellow dot indicates the location of IRT/TLS acquisitions from El Capitan Meadow. Background relief map: 1 m × 1 m cell size aerial laser scanning digital elevation model. (**b**) 3-D photorealistic model of the southeast face of El Capitan (high-resolution TLS mesh textured with a gigapixel panorama acquired in October 2015). (**c**) 3-D geological model of the southeast face of El Capitan (high-resolution TLS mesh textured with a vertical geologic map^79^). (**d**) Boot Flake and Texas Flake area framed in red in Panels *b* and *c*. (**e**) High-angle view of Texas Flake that shows the back face of the flake; photographic credit: 2019 Google, El Capitan Street View Trek. The yellow arrow indicates the location of several chockstones within the rear fracture. (**f**) Detail view of the rear fracture in the area of the yellow arrow in Panel *e*. The image was overexposed to better visualize the chockstones. The uppermost chockstone (shown by the arrow) is located 8.5 m below the top of Texas Flake (see Supplementary Fig. 2).

Our study focused on the vertical-to-overhanging granitic rock wall of El Capitan, located in Yosemite Valley, California, USA, within the central Sierra Nevada (Fig. 1a,b). Over the past 150 years, rockfalls have been intermittently documented^9^ from the southeast face of El Capitan, with 45 rockfalls recorded^6,71^ between 1857 and 2017. Exfoliation sheets are characteristic of the Yosemite landscape^72,73^, and are ubiquitous on El Capitan. Boot Flake and Texas Flake are particularly impressive exfoliation sheets (Fig. 1d,e) and are famous features given their location along the popular rock climbing route “The Nose”.

The cliff face of El Capitan was initially exposed by glacial erosion (and later, by rockfalls^72,74^) into late-Cretaceous plutons of the Yosemite Valley Intrusive Suite that is part of the greater Sierra Nevada Batholith^75,76^. These plutons are mainly composed of granite and granodiorite, with lesser quantities of diorite, tonalite, aplite and pegmatite^73,75,77–80^. Two main geologic units form the majority of the southeast face of El Capitan (Fig. 1c): El Capitan Granite (Kec, 106.1 ± 0.1 Ma) and Taft Granite (Kt, 106.6 ± 0.7 Ma)^76,77,79–81^. These rocks were intruded by the diorite of North America (Kd, 104.0 ± 0.4 Ma) and Leaning Tower Granite (Klt, 104.1 ± 0.1 Ma)^76,77,79–81^. Other geologic units forming the southeast face of El Capitan include the tonalite of the Gray Bands (Kgb) on the upper part of the southeast face (Fig. 1c), as well as two types of dikes (aplite dikes (Kap) and dikes of the Oceans (Kdo) along with some pegmatite^76,77,79–81^. The exfoliation sheets forming Boot Flake and Texas Flake, which are the focus of our study, consist of Kec with some thin intrusions of Kap and Kdo (Fig. 1d). These two sub-vertical flakes are relatively flat and have almost the same spatial orientation (mean dip direction: 153°; mean dip angle: 81°) on the cliff. The respective dimensions of Boot Flake and Texas Flake (measured on TLS data) are: maximum length: 17.4 m and 26.4 m; maximum width: 5.1 m and 11.3 m; mean width: 3.9 m and 7.8 m; mean thickness: 25 cm and 72 cm; and mean aperture: 8 cm and 33 cm.

## Materials and Methods

Our research focused on using thermal imaging technology to identify warm (attached) and cool (detached) parts of exfoliation sheets to quantify rock bridge area for quantitative rockfall stability analyses. Here, we outline our methods of thermal panorama image data acquisition. We subsequently used numerical modeling to calibrate and confirm our image-based findings.

### Thermal imaging

#### Data acquisition and technical specifications of the thermal camera

**Figure 2:**
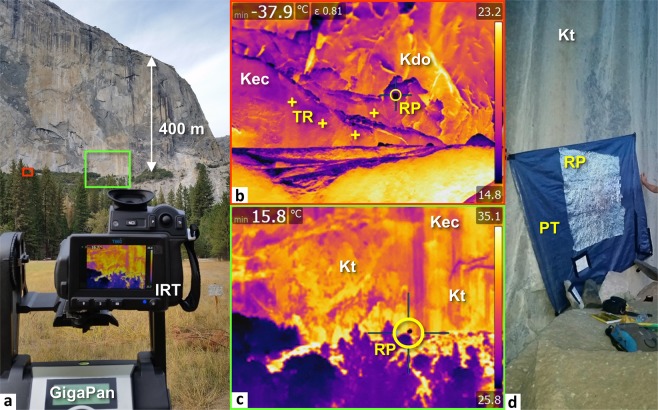
Experimental IRT measurement setup. Key to abbreviations: Kec = El Capitan Granite; Kdo = dikes of the Oceans; Kt = Taft Granite; PT = Plastic tarpaulin; RP = Reflective paper; TR = Thermoresistance. (**a**) Front view of the southeast face of El Capitan from El Capitan Meadow. The foreground shows the FLIR T-660 infrared thermal camera (IRT) mounted on a GigaPan EPIC Pro robotic system. In the background, the green frame shows the camera’s field of view and the red frame shows the area that was chosen to measure the emissivity of Kec. (**b**) Thermogram taken at the cliff foot (sector framed in red in Panel *a*) during the determination of emissivity, ε of Kec (time shown is 8 October 2015 at 01:36 PST). The four yellow crosses indicate the position of the Pt100 sensors (TR) used to adjust the emissivity. After calibration, the reflected apparent temperature of the aluminum foil (RP) was −37.9 °C (coldest pixel) at 01:36 PST. For these proximity measurements, the reflective paper was eight times smaller than the one visible in Panel *d*. The temperature scale on the right side of the panel is in degrees Celsius. (**c**) Thermogram displayed by the thermal camera on 8 October 2015 at 16:54 PST in the area framed in green in Panel *a*. Before calibration, the reflected apparent temperature of the aluminum foil (RP) was 15.8 °C (coldest pixel). The temperature scale on the right side of the panel is in degrees Celsius. (**d**) Photo taken at the cliff foot during the installation of the large reflective paper (area circled in yellow in Panel *c*). The 2 m^2^ plastic tarpaulin (PT) was half covered with crumpled, then uncrumpled, aluminum foil (RP).

We acquired thermal images (thermograms) of the southeast face of El Capitan using a FLIR T-660 thermal imager (IRT camera) mounted on a GigaPan^82^ EPIC Pro robotic device (Fig. 2a). We collected data during the evening of 8 October 2015 beginning at 17:45 Pacific Standard Time (PST); October is one period in which maximum thermal changes to rock faces have been documented to occur in previous studies of rock exfoliation in Yosemite^12^. To increase the resolution of the data in specific areas of interest (Boot Flake and Texas Flake), we monitored these areas (one thermogram every 20 minutes) between 18:20 and 21:40 PST using a fixed tripod for the IRT camera. The FLIR camera has an infrared resolution of 640 × 480 pixels and is equipped with a 5-million-pixel digital RGB camera. The camera possesses a field of view of 25° × 19°, a focal length of 25 mm, an infrared spectral range of 7.5–14 µm, and a temperature measurement range of −40 °C to +2,000 °C with an accuracy^83^ of ±1 °C. The thermal sensitivity (or thermal resolution) is quantitatively defined by the Noise Equivalent Temperature Difference (NETD)^70,84^, which represents “*the difference between the temperature of an observed object and the ambient air temperature that generates a signal level equal to the noise level*”^85^. The lower the NETD, the more the camera is able to distinguish small thermal contrasts. For the FLIR T-660 imager, the NETD is less^83^ than 0.02 °C at 30 °C.

#### Thermographic calibration protocol

The temperature of a target object measured by an IRT camera depends on its emissivity and is affected by direct radiation reflected by the object (Supplementary Fig. 1). Emissivity (which varies between 0 and 1) is defined by the ratio between the radiant energy emitted by the surface of the measured object and that emitted by a perfect blackbody under the same conditions (temperature, direction of observation, and wavelength)^86–88^. For low-emissivity materials, it is essential to accurately measure this value because small variations in emissivity can lead to large variations in the corrected temperatures^83,89^. The temperature of an object is also influenced by radiation from both the camera and object environment^57,83,90^ (Supplementary Fig. 1). To correct for these artifacts and obtain temperature values close to the real surface temperatures of the object, it is mandatory to calibrate the apparent temperatures measured by the camera. This is performed by specifying several parameters including the emissivity of the object, the reflected apparent temperature, the ambient air temperature and relative humidity, and the distance between the target and the camera^83,89^.

#### Determination of calibration parameters

We calibrated the emissivity of El Capitan Granite at the foot of the southeast cliff face from reference temperatures given by four platinum resistance temperature detectors (Pt100 type sensors with accuracy^91^ of ±0.1 °C at 20 °C) fixed to the rock (Fig. 2b). With the data from these sensors, along with the reflected apparent temperature, ambient air temperature, and relative humidity (presented subsequently), we calibrated the thermal camera to find the reference temperatures on the thermogram, which provided a mean emissivity value of 0.81.

To measure the reflected apparent temperatures from El Capitan Meadow, we fixed reflective paper at the base of the cliff (Fig. 2d) to obtain a reflective signal (i.e., diffuse reflector method^83,88,89,92^). Although we were positioned more than 1 km from the rock wall (Fig. 2a), the reflective paper was accurately detected with the IRT camera (Fig. 2c). The temperature reading directly provides the value of the reflected apparent temperature (Table 1).

**Table 1:** Calibration parameters measured during the IRT monitoring of 8 October 2015.

Time of thermal image PST	Reflected apparent temperature °C	Ambient air temperature °C	Relative humidity %
17:45	18.6	23.1	39
18:20	15.3	20.3	50
18:40	14.1	18.2	56
19:00	13.8	15.8	63
19:20	13.2	14.9	68
19:40	10.4	14.1	73
20:00	7.3	11.4	83
20:20	9.7	11.2	79
20:40	9.5	10.9	85
21:00	8.0	9.5	94
21:20	9.2	10.3	85
21:40	7.4	11.8	85

Ambient air temperature and relative humidity affect the transmission of infrared radiation (i.e., the atmospheric transmission factor^70,87,88^). We measured these parameters using a pocketsize digital thermohydrometer (measurement resolution^93^ of 0.1 °C and 1% for temperature and relative humidity, with accuracy^93^ of ±1 °C and ±3% between 30% and 80% relative humidity (±5% outside this range), respectively). To calibrate temperatures at different acquisition times, we measured the reflected apparent temperature, relative humidity and ambient air temperature before each acquisition.

The final parameter needed for calibration of the thermographic data is the distance between the camera and the object of interest. We obtained this data using an Optech ILRIS-LR terrestrial lidar system (TLS) located at 1,037 m distance (as measured between the thermal camera in El Capitan Meadow (Fig. 1a) and the aluminum foil reflector). Our TLS device (which we also used to collect a 3-D point cloud of the southeast face of El Capitan on 7 October 2015) has a manufacturer-specified accuracy^94^ of 7 mm at a range of 100 m. Our resultant point cloud is composed of about 38 million points with a mean point spacing, equivalent to the spatial resolution, of 15 cm. We georeferenced the point cloud data onto an existing 1 m aerial Digital Elevation Model (DEM) of Yosemite Valley using Iterative Closest Point (ICP) algorithms^95,96^ implemented in CloudCompare^97^ software. The georeferenced point cloud allowed us to extract the geometry, spatial orientation, and dimensions of Boot Flake and Texas Flake, located at a distance of 963 m and 938 m, respectively from the IRT camera.

#### Post-processing and registration of thermal images

We generated the final El Capitan thermal panorama by stitching 30 individual thermal images using Kolor^98^ Autopano Giga software. To correct the shifts of some pixels due to the handling of the camera when specifying updated calibration parameters (i.e., when monitoring Boot Flake and Texas Flake), we exported all IRT images with the same temperature scale and co-registered these using roto-translations. We used the function *imregister* (part of the *Image Processing Toolbox*^99^) to automatically align images of the same size from the *Intensity-based automatic image registration* algorithm in MATLAB. Thus, each image at *t*_i+1_ was aligned on the image at *t*_*i*_ so as to avoid abrupt intensity (temperature) changes between two acquisitions. Ultimately, our registration process achieved sub-pixel (<0.75 m at 950 m distance) accuracy.

### Thermal modeling

To demonstrate the link between rock bridge existence and warm thermal anomalies, we performed 2-D thermal modeling using COMSOL^100^ Multiphysics® software. Here, we present only the modeling of the thermal behavior of the lower part of Boot Flake for brevity.

#### Geometry and physical properties of the material

**Figure 3:**
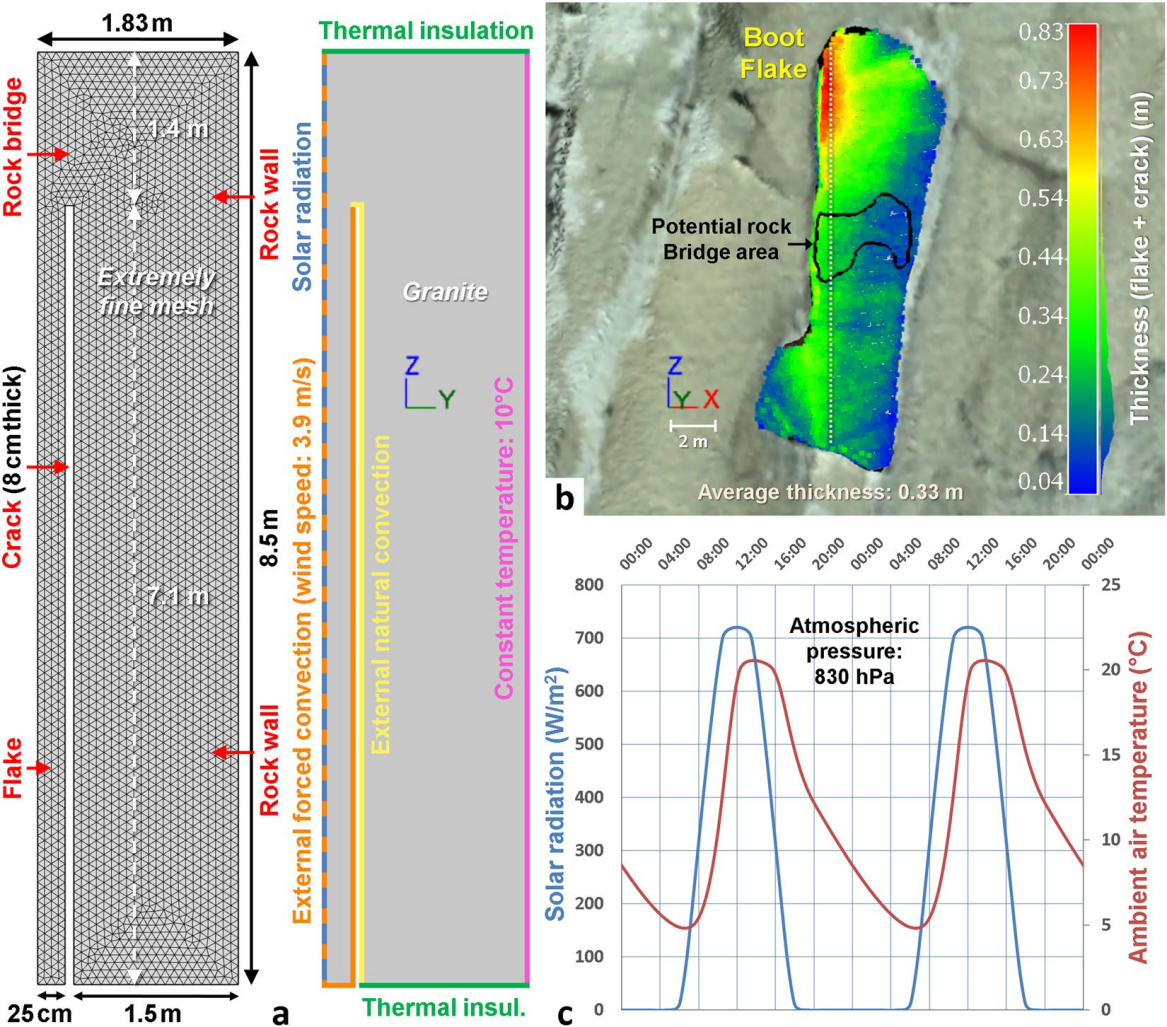
Properties of 2-D thermal modeling of the lower half of Boot Flake. (**a**) Geometry, meshing condition (left) and boundary conditions of the model (right). (**b**) Point-to-mesh deviations derived from the TLS data showing the thickness between the rock wall and the flake surface. The 33 cm corresponds to the average thickness along the white dashed line where the temperature profiles of Figs 5d and 6b have been established. The black contour shows the location of the potential rock bridge of Boot Flake. This contour delineates the maximum surface area of the warm thermal anomaly visible in the center of Boot Flake (see Fig. 7a,b). (**c**) Warming and cooling cycles applied to the modeled flake from solar radiation (blue) and ambient air temperature (red).

We determined the geometry of Boot Flake based on the TLS model and a profile photograph^101^ of the east side of the flake taken by rock climbers. The flake is approximately 17 m length with a total surface area of 75.5 m^2^. Given the overall symmetry of the flake (the lower west “toe section” notwithstanding), we modeled only the lower half of the flake (with area of 37.8 m^2^ and length of 8.5 m; Fig. 3a). In accordance with measurements performed on temperature profiles and 3-D “thermo-photorealistic” models (see the third section of the results), a length of 1.4 m was attributed to the rock bridge. We estimated the thickness of the flake to be 25 cm based on relative measurements from the profile picture. From the TLS model, we measured the average distance between the rock wall and the flake surface to be 33 cm (as measured along the vertical profile in Fig. 3b). Thus, we assigned an aperture of 8 cm for the crack. In addition, we assigned a 1.5 m thickness for rock wall in the model in order to generate a sufficiently large thickness contrast with the flake.

The intrinsic properties of the granite (Table 2, and assumed homogenous and isotropic) chosen for 2-D thermal modeling come from the COMSOL Multiphysics® software’s integrated material library. Only the emissivity was added to the basic properties and only the thermal conductivity was adjusted from 2.9 to 2.2 W/(m∙K) in order to obtain lower surface temperatures, and thus closer to the *in situ* measured values.

**Table 2:** Physical properties of granite used for 2-D thermal modeling.

Thermal conductivity W/(m∙K)	Heat capacity at constant pressure J/(Kg∙K)	Emissivity	Density g/cm^3^
2.2	850	0.81	2.60

#### Heating-cooling cycles applied to the 2-D model

To obtain a realistic temperature field for the Boot Flake model, we applied several daily heating and cooling cycles (twelve in total) to the model, coincident with standard modeling practices e.g.,^102,103^. The flake surface temperature and temperature depth propagation were calculated at 5-minute intervals. We estimated solar radiation using the methodology of Duffie and Beckman^103^ and with a declination based on Cooper’s equation^104^. In mid-October, and for a south-southeast exposure at 1,740 m, the solar radiation is zero between 18:30 and 05:30 PST and reaches its maximum value (725 W/m^2^) at 12:00 PST (Fig. 3c). We modeled ambient air temperature fluctuations with a pseudo-sinusoidal curve function varying between 4.8 °C (06:30 PST) and 20.8 °C (13:30 PST). These values are based on temperature data from a nearby weather station^105^ at Yosemite National Park Headquarters, located at Yosemite Village (Fig. 1a). The initial temperature of the entire geometry was set at 10.0 °C at 06:30 PST (t = 0; beginning of the warming cycle) such that it is equal to the constant temperature fixed at the inner limit of the rock wall (Fig. 3a,c). At 06:30 PST, the solar radiation affecting the outer flake surface is 80 W/m^2^ (Fig. 3c) and for this simulation, we considered the atmospheric pressure as constant and equal^106^ to 830 hPa at an elevation of 1,700 m.

The geometry defined in Fig. 3a requires imposing thermal insulation conditions on its upper and lower edges to take into account the symmetry of Boot Flake and the extension of the rock wall, respectively. The insulation condition was not applied to the lower end of the flake where it is in contact with air (Fig. 3a). The influence of air is modeled by convective heat fluxes that take into account the ambient air temperature, the inclination angle, and the length of the surface on which the condition applies. In our model, we use two types of convection: (1) external natural convection (in yellow in Fig. 3a); and (2) external forced convection (in orange in Fig. 3a). The first, induced by a gradient of temperature, is applied at the crack level along the outer edge of the rock wall and the lateral edge of the rock bridge. The second, produced by a wind circulation (taken as constant at 3.9 m/s to obtain lower, and more realistic surface temperatures) affects the entire flake and allows more efficient cooling.

## Results

### Thermal signatures of El Capitan geologic units

**Figure 4:**
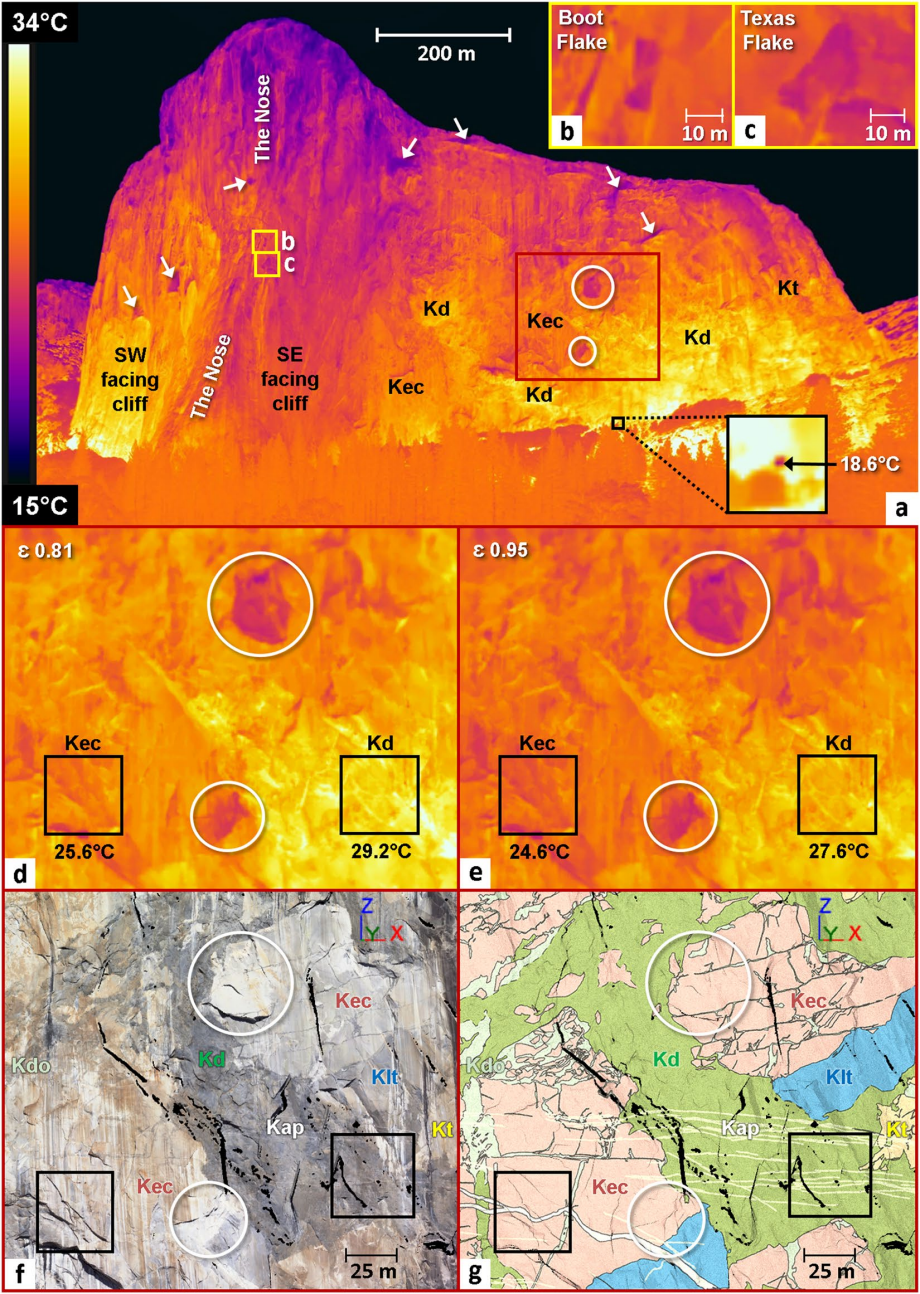
Classification of thermal signatures of El Capitan geologic units. Key to abbreviations: Kap = aplite dikes; Kec = El Capitan Granite; Kd = diorite of North America; Kdo = dikes of the Oceans; Klt = Leaning Tower Granite; Kt = Taft Granite; ε = Emissivity. (**a**) IRT panorama (2820 × 1525 pixels –30 stitched thermal images) of the southeast face of El Capitan on 8 October 2015 at 17:45 PST. The black frame shows the position of the reflective paper as well as the resulting reflective apparent temperature (18.6 °C; coldest pixel) measured after calibration. The white circles and white arrows highlight cold thermal anomalies that correspond respectively to lighter-colored rock areas and overhanging areas. (**b**) Detail view of Boot Flake framed in yellow in Panel *a*. (**c**) Same as Panel *b* but for Texas Flake. (**d**) Detail view of the cliff area framed in reddish-orange in Panel *a*. The average temperature of each black frame (50 × 50 pixels) is indicated under the frames. (**e**) Same as Panel *d* but with an emissivity ε of 0.95. (**f**) Extract from the 3-D photorealistic model of Fig. 1b corresponding to the cliff area framed in reddish-orange in Panel *a*. (**g**) Extract from the 3-D geological model of Fig. 1c corresponding to the cliff area framed in reddish-orange in Panel *a*.

The IRT panorama of El Capitan (Fig. 4a) acquired on 8 October 2015 at 17:45 PST shows calibrated temperatures ranging between 33.1 °C at the base of the cliff and 15.8 °C at the top. Thus, we observe an increase of 17.3 °C for a 950 m increase in elevation (i.e., +1.8 °C/100 m). Given the average adiabatic lapse rate for a dry atmosphere is around 2.0–3.0 °C for every 300 m increase in elevation^107^, this temperature range is two to three times higher than expected for neutral surroundings. This variance is due to the different incidence angles of infrared thermal radiation (Supplementary Fig. 1); the top of El Capitan reflects a portion of the colder sky (typical temperature of −60 °C on a thermal camera^108^) and the base of the cliff reflects a portion of the warmer ground (talus slope, vegetation). Thus, the minimum and maximum temperatures measured on El Capitan are overestimated (i.e., too low and too high, respectively). The coldest temperatures (<19.0 °C) are located in the upper part of cliff and correspond to southeast-facing rock faces that are in shadow beginning in the mid-afternoon in October. The warmest thermal signatures (>30.0 °C) occur at the base of the cliff, directly above southwest-facing talus slope areas that are heated until the late afternoon. For the remainder of the rock wall, the majority of the coldest zones correspond either to overhangs and southeast-facing facets or to lighter-colored rock areas. The data (Fig. 4d through 4g) show that temperatures also vary depending on lithology. For example, Kd (a dark-colored rock; Fig. 4f) displays higher temperature values (by about 3.6 °C) compared to Kec (a light-colored rock; Fig. 4f). However, this temperature deviation should be interpreted with caution because their different color implies different emissivities. By selecting a more appropriate emissivity value to the darker color of the Kd lithology (i.e., 0.95; measured value for the Glacier Point Granodiorite, a rock type outcropping to the east of Yosemite Village which shows similarities (color, structure) with Kd), the average temperature difference between these two rock types is 2.0 °C (Fig. 4e). Thus, the difference in emissivity alone does not explain the thermal contrast highlighted between Kec and Kd; the texture and mineralogical composition of the rock must therefore play a significant role. In summary, the three main factors that explain the observed thermal signatures are: (1) the incidence angle of infrared thermal radiation (related to the viewing angle of the camera); (2) the geometric features of the cliff (overhangs, dihedrals, buttresses, etc.); and (3) the emissivity and color of the different rock types.

The thermographic calibration protocols allow global corrections that mitigate the effect of these artifacts on the thermograms. However, further geometric corrections to the whole panorama are required to obtain a more realistic vertical temperature gradient. Thus, to learn more about the thermal signature of rockfall-prone exfoliation sheets (i.e., at a much smaller scale compared to the entire face of El Capitan), we focused our monitoring on the Boot Flake and Texas Flake area (Fig. 4b,c) where the incidence angle can be considered as constant, and the geometry and rock types show only minor variations. Thus, if thermal anomalies are visible at the scale of several hundred meters, the most interesting ones, in the aim of detecting potential rock bridges, should be coincident with the local anomalies.

### Detection of potential rock bridges for Boot Flake and Texas Flake

**Figure 5:**
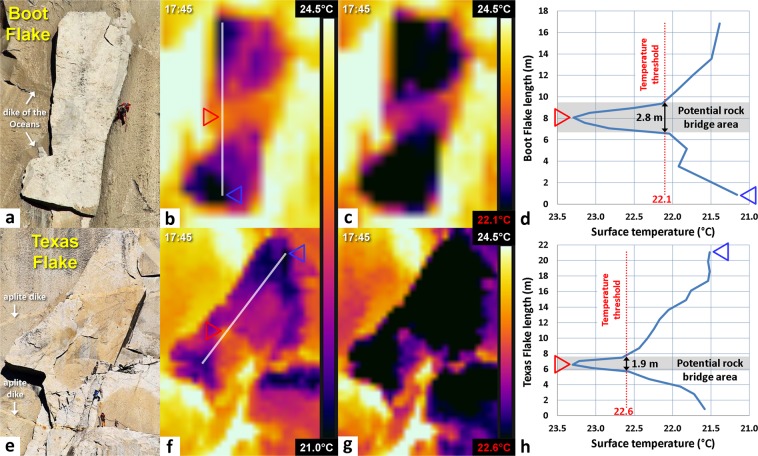
Thermal signature of Boot Flake and Texas Flake on 8 October 2015 at 17:45 PST. (**a**) Front view of Boot Flake with a climber on the Nose climbing route; photographic credit: Thomas M. Evans (photograph reproduced under the CC BY 4.0 license). (**b**) Thermogram of Boot Flake with a temperature scale ranges from 21.0 °C to 24.5 °C. The red and blue triangles indicate the highest (23.3 °C) and lowest (21.2 °C) temperatures, respectively measured on the white longitudinal profile. The temperature profile passes through 20 pixels. (**c**) Same as Panel *b* but with a temperature scale ranges from 22.1 °C to 24.5 °C. (**d**) Temperature profile along Boot Flake. The location of this profile is shown in Panel *b*. The dotted red line is drawn at the greatest slope break of the profile and indicates the value (22.1 °C) of the temperature threshold used in Panel *c* to reveal the temperatures outside the potential rock bridge area (shown in black). (**e**) Front view of Texas Flake with climbers on the Nose climbing route; photographic credit: Thomas M. Evans (photograph reproduced under the CC BY 4.0 license). (**f**) Thermogram of Texas Flake with a temperature scale ranges from 21.0 °C to 24.5 °C. As in Panel *b*, the red and blue triangles indicates the highest (23.3 °C) and lowest (21.5 °C) temperatures, respectively measured on the white longitudinal profile. The temperature profile passes through 32 pixels. (**g**) Same as Panel *f* but with a temperature scale range from 22.6 °C to 24.5 °C. (**h**) Temperature profile along Texas Flake. The location of this profile is shown in Panel *f*. The dotted red line indicates the slope break value (22.6 °C) of the temperature threshold used in Panel *g* to reveal the temperatures outside the potential rock bridge area (shown in black).

**Figure 6:**
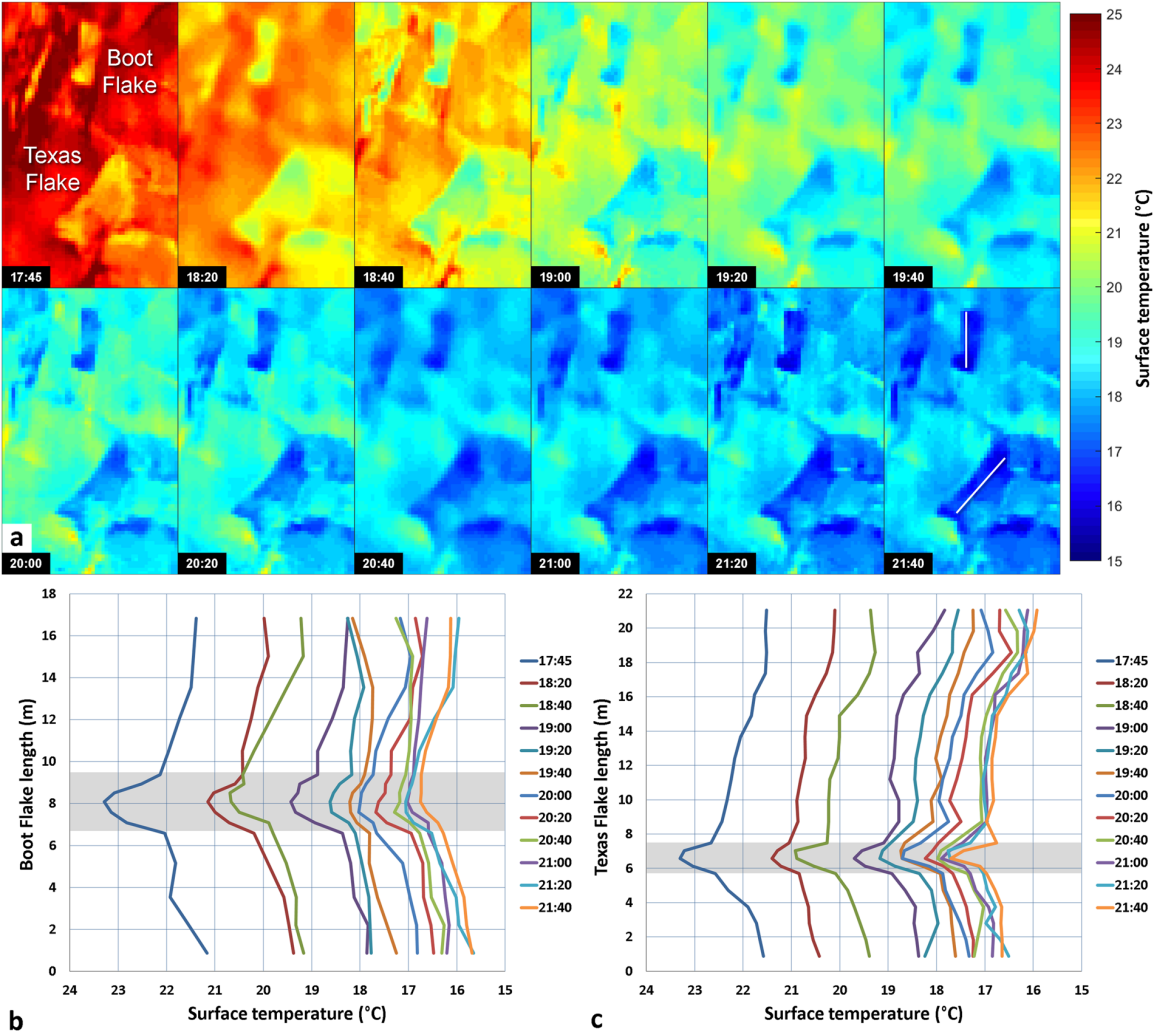
Results of the IRT monitoring performed on 8 October 2015 in the Boot Flake and Texas Flake area. (**a**) Sequence of the thermograms acquired between 17:45 and 21:40 PST. The last thermogram shows the location of the two temperature profiles defined in Fig. 5b,f. (**b**,**c**) Evolution of the surface temperatures along longitudinal profiles of Boot Flake and Texas Flake between 17:45 and 21:40 PST. The shaded areas coincide with those defined in Fig. 5d,h and correspond to the potential rock bridge areas of each flake.

The most notable observation from the Boot Flake and Texas Flake thermal data are that the flakes always remain colder than the surrounding cliff areas (Figs 5 and 6) between 17:45 and 21:40 PST (i.e., during a cooling sequence). In addition, they display non-homogeneous temperature patterns: a warm thermal anomaly is visible in the center of Boot Flake (Figs 5d and 6b) and in the lower part of Texas Flake (Figs 5h and 6c). Although the bell-shaped thermal anomaly in Boot Flake becomes less pronounced over time, it remains visible on every temperature profile. The best thermal contrast occurs at 17:45 PST and is better visualized using a temperature scale ranging from 21.0 °C to 24.5 °C. At that time, the coldest part of the lower edge of Boot Flake (blue triangle in Fig. 5b) and the warmest adjacent area outside the flake (yellow area) present a difference of temperature of 3.6 °C. For Texas Flake, the coldest portion is located on its upper edge (Fig. 5f); the temperature difference here is 2.6 °C. At the flake surface, both warmer thermal anomalies indicate a maximum temperature of 23.3 °C (red triangles), which correspond to respective deviations of 2.1 °C and 1.8 °C with the blue triangles of Boot Flake and Texas Flake (Fig. 5d,h).

**Figure 7:**
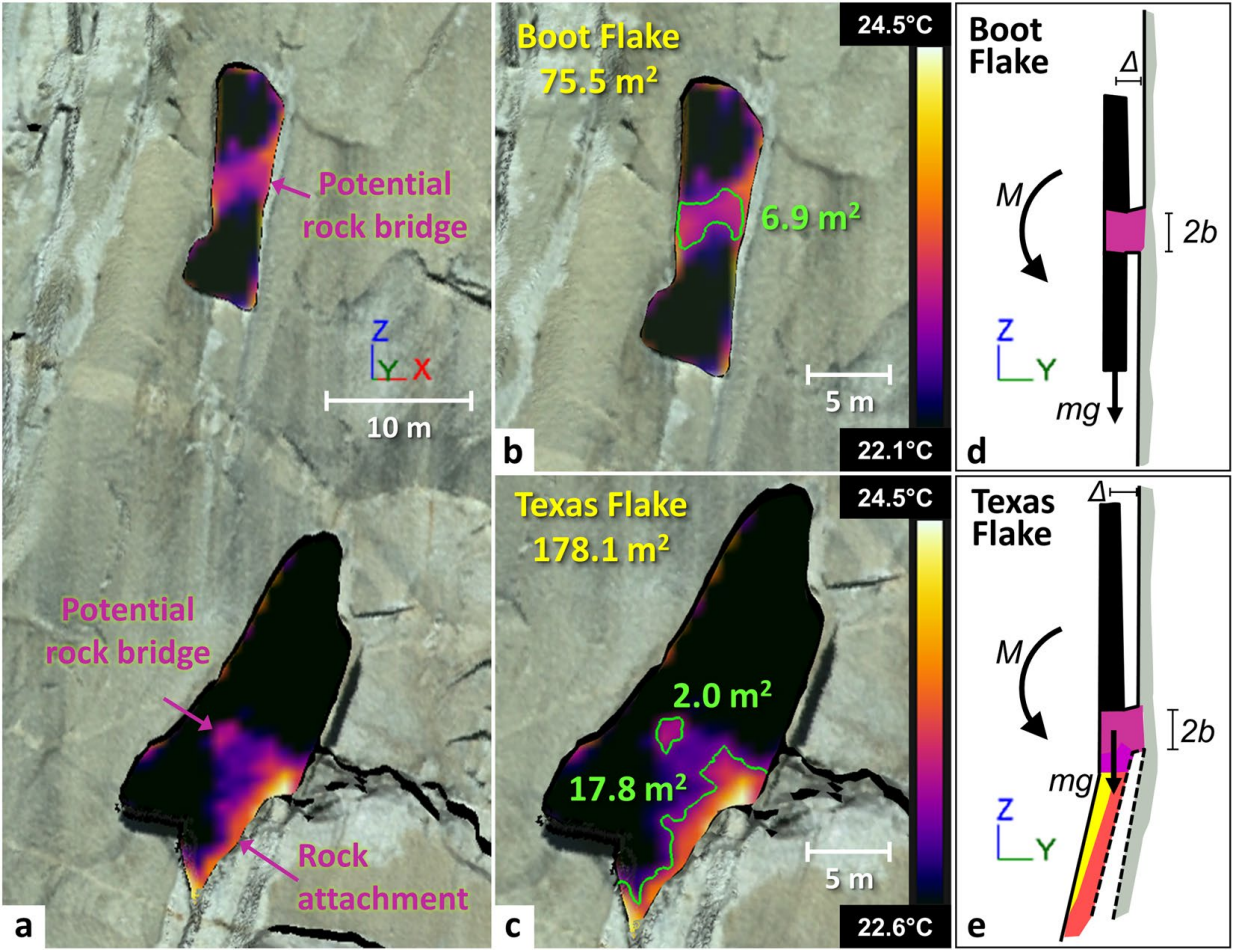
3-D “thermo-photorealistic” model of the Boot Flake and Texas Flake area. (**a**) High-resolution TLS mesh textured with a gigapixel panorama acquired in October 2015 and both thermograms from Fig. 5c,g. (**b**) Detail view of Boot Flake. The green 3-D polyline delineates the maximum surface area of the warm thermal anomaly that could be associated with the existence of a rock bridge. (**c**) Detail view of Texas Flake. Both green 3-D polylines delineate the maximum surface areas of the two warm thermal anomalies. The uppermost polyline could be associated with the existence of a rock bridge while the lower one corresponds to an area labeled “rock attachment”. Unlike the rock bridge, the rock attachment is not delimited on all sides. (**d**,**e**) Cross-section schematic views of Boot and Texas flakes. The geometry and acting stresses (self-weight, *mg*, acting at a distance *Δ* to create a tensile moment, *M*), that acts to rotate the flakes away from the cliff across an attachment point with radius, *b*, refer to Eq. 1.

We favor rock bridges connecting the flakes to the cliff as the source of the warm thermal anomalies because other explanations (e.g., rock type variations, chockstones wedged behind the flakes) do not account for the observations. In terms of location, Boot Flake’s central anomaly coincides approximately with the position of a dark circular dike (Dike of the Oceans) situated at the same height (Fig. 5a). However, this dike only partially crosses the west side of the warm anomaly. The absence of a structure or color change on the east side of the flake favors the presence of a rock bridge in this area (at least on the east side). In the bottom part of Texas Flake, the lack of a dike that might be invoked to account for the thermal anomaly strengthens the rock bridge hypothesis suggested for Boot Flake. Additionally, Fig. 1e shows that Texas Flake must be held to the rock wall at its base by an area that we called “rock attachment” (unlike the rock bridge, this area is not delimited on all sides). Nevertheless, the data suggests that the warm thermal anomaly of Texas Flake defines two parts of potential attachment given the decrease and then increase in warm signal moving up and to the west from the rock attachment area. Thus, to the west of this zone, we assume the existence of a small rock bridge where the temperature profile has been drawn (Fig. 7c −2.0 m^2^). This assumption is reinforced by the knowledge that the top of this potential rock bridge is 13.9 m below the top of Texas Flake at 1,699.2 m (Supplementary Fig. 2), i.e. well below the position of the uppermost chockstones (8.5 m below the top of Texas Flake) highlighted in Fig. 1f. The chockstones do not appear as thermal anomalies due likely to the cooling that can occur around the chockstones behind the flake - the ventilation is sufficient to prevent a detectable thermal bridge.

The non-homogeneous temperature pattern is explained more rigorously by consideration of the cooling that is possible behind the flakes due to air circulation. In the area of a potential rock bridge, the rock would conduct heat into the flake from the cliff behind. Meanwhile, air circulation in the fracture between the flake and the cliff would result in lower temperatures in the flake away from the rock bridge.

### Dimensions of potential rock bridges

Given our hypothesis that the warm thermal anomalies are where the flakes are still attached to the cliff, we used a combination of thermal imaging and TLS to determine the surface areas of potential rock bridges. By measuring the distance between the greatest slope breaks on the temperature profiles (Fig. 5d,h), we obtained preliminary maximum rock bridge lengths of 2.8 m and 1.9 m for Boot and Texas flakes, respectively. We subsequently used the temperatures (22.1 °C for Boot Flake and 22.6 °C for Texas Flake) associated with the plot position of these slope breaks to determine the minimum temperatures (or temperature thresholds) to be applied to the thermograms to display only a color contrast within warm thermal anomalies. Thus, all portions of the flake with temperatures below the temperature thresholds (i.e., where the rear fracture is open) appear in black on the thermograms (Fig. 5c,g). This procedure allows better delimitation of the external contours (individualization) of the warm thermal anomalies. By draping these “thresholded thermograms” on the TLS data, we estimated the maximum surface area (6.9 m^2^ and 2.0 m^2^) of the inferred rock bridges for Boot and Texas flakes, respectively (Fig. 7a to 7c). Remotely identifying the area of potential attachment of rock bridges holding the flake onto the wall while still attached is an achievement never before quantified in this detail or with this methodology.

### Modeled temperature profiles versus measured profiles

**Figure 8:**
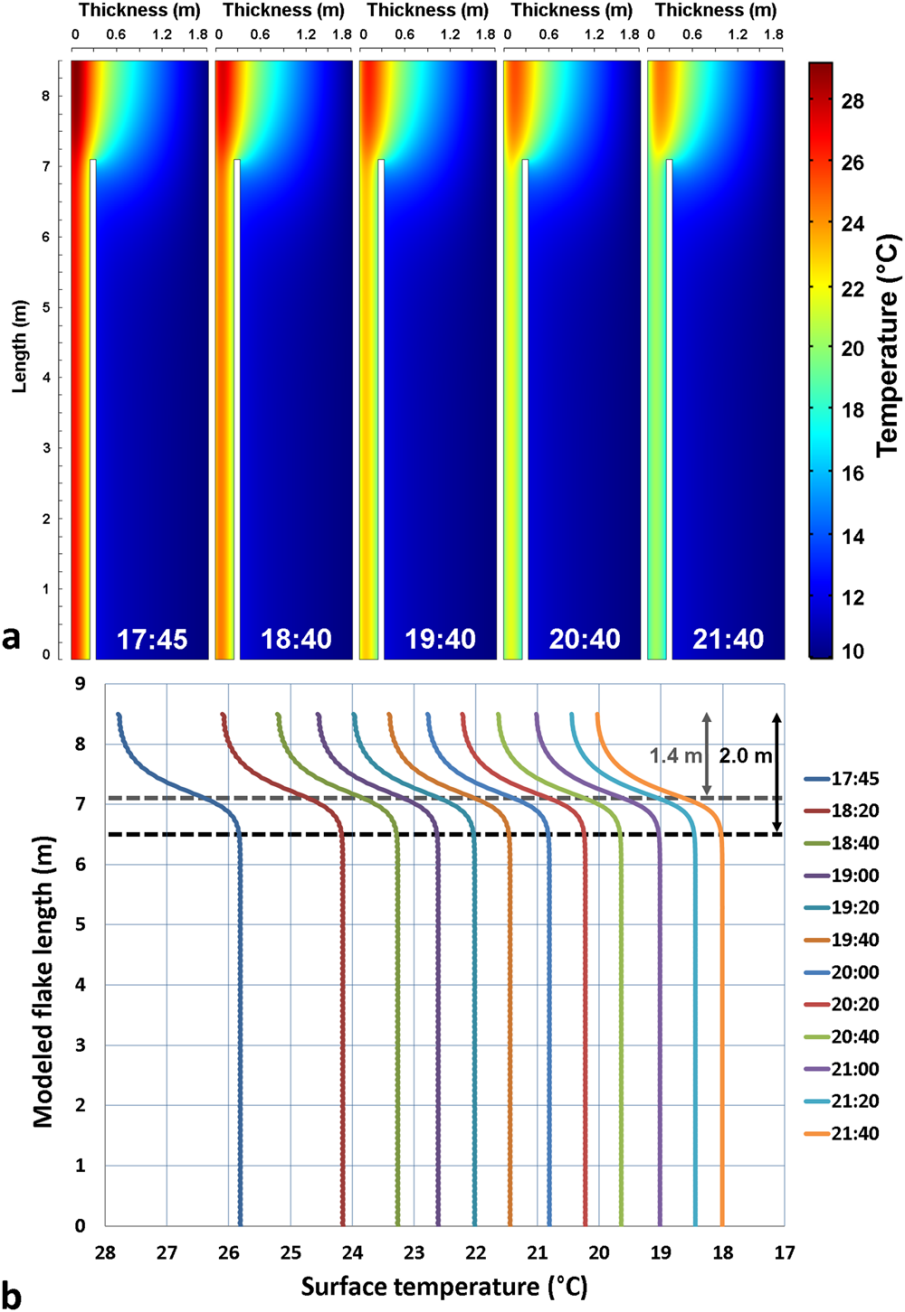
Results of 2-D thermal modeling of the lower half of Boot Flake for a 1.4-m-long rock bridge (results of the 12^th^ and final day of simulation). (**a**) Hourly temperature evolution within the model between 17:45 and 21:40 PST. (**b**) Evolution of the surface temperatures (rock depth = 0 m) along the modeled flake between 17:45 and 21:40 PST. The gray and black dashed lines indicate the respective lengths of the rock bridge (1.4 m) and the warm thermal anomaly (2.0 m). The times displayed for the profiles are the same as those from the IRT monitoring.

At the end of the twelve simulated daily heating and cooling cycles, the results of the 2-D thermal modeling of the lower part of Boot Flake are similar to those of the IRT monitoring, namely that the rock bridge is always warmer than the detached part of the flake during nocturnal rock cooling (Fig. 8a). Although the modeled surface temperatures are, on average, 4 °C higher than those measured (resulting from several potential sources, including the use of generalized, rather site-specific, modeling parameters – Table 2; see also the Discussion section), all the profiles show a bell-shaped anomaly at the rock bridge position (Fig. 8b). This result therefore confirms our hypothesis on the presence of a rock bridge in the center of Boot Flake. However, the thermal modeling also shows that the length of the warm thermal anomaly (2.0 m) is greater than that of the rock bridge (1.4 m) (Fig. 8b). Consequently, the previously estimated dimensions (length, surface area) of the potential rock bridges are overestimated.

**Figure 9:**
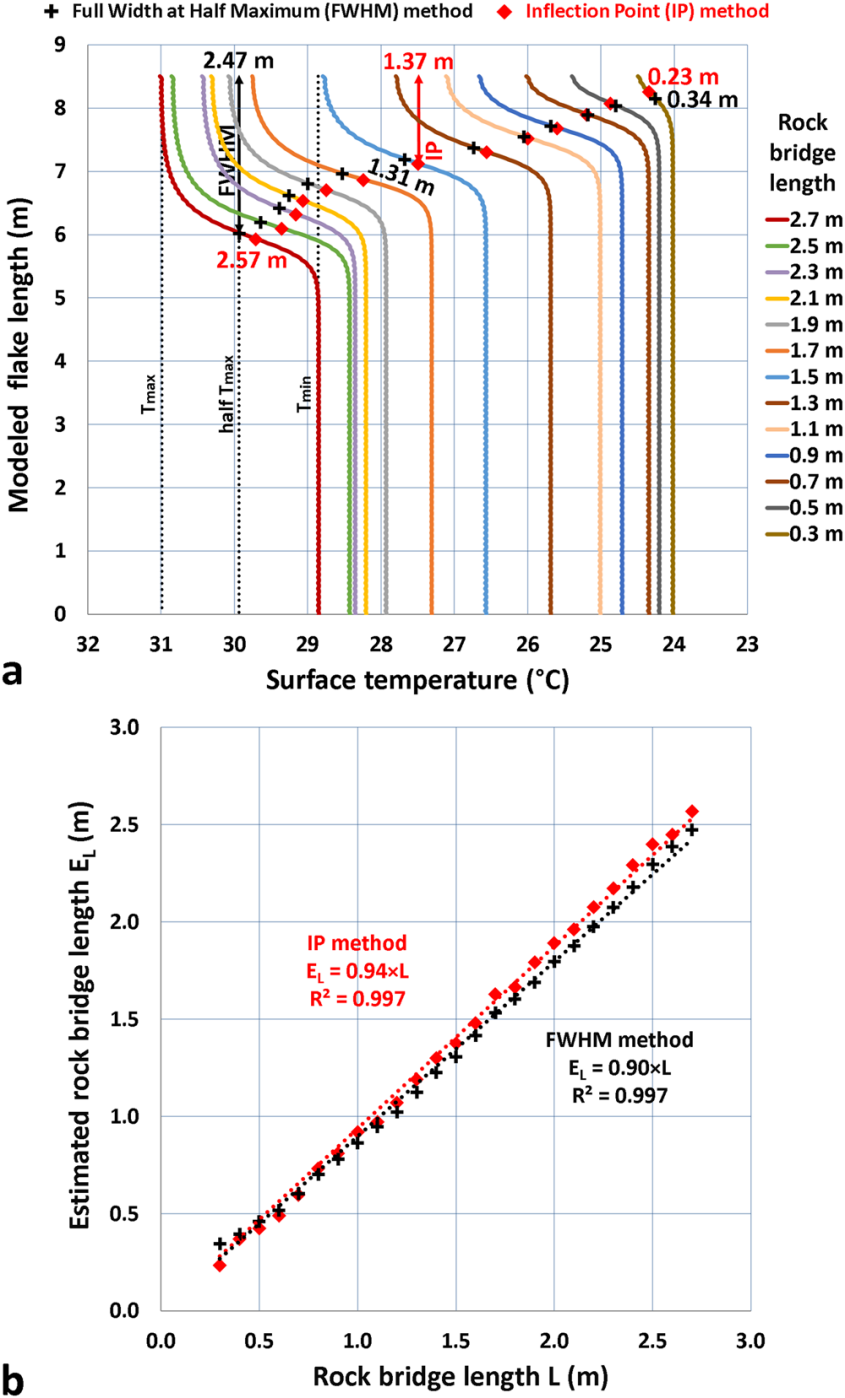
Results of 2-D thermal modeling of the lower half of Boot Flake for different lengths of rock bridges (results of the 12^th^ and final day of simulation at 17:45 PST). (**a**) Temperature profiles obtained on the surface (rock depth = 0 m) of the modeled flake for rock bridge lengths ranging between 0.3 and 2.7 m. To avoid overloading this graph, only 13 out 27 rock bridge profiles are displayed; here, the rock bridge length increases by 0.2 m at each profile. The black crosses indicate the position of the Full Width at Half Maximum (FWHM) for each profile, whereas the red diamonds indicate the position of the Inflection Point (IP) for each profile. The method for determining the FWHM is shown for the profile obtained with a 2.7-m-long rock bridge; it is given by the distance at which the profile reaches half its maximum value. For this profile, the FWHM method estimates the rock bridge length at 2.47 m. The red diamonds indicate the position of the inflection point for each profile. The distance passing through this point gives a second “statistical” estimate of the rock bridge length. (**b**) Relationship between the modeled length (L) and the estimated length (E_L_) of the rock bridges. The estimated lengths correspond to all the values determined with the FWHM and IP methods; six of these values are specified on Panel *a*.

Based on this finding, we then tested two simple slope-break profile methods to try to find the “true” size of the rock bridges from the temperature profiles: (1) the Full Width at Half Maximum (FWHM) method; and (2) the Inflection Point (IP) method. The FWHM is given by the distance at which the profile reaches half its maximum value whereas the IP method gives the distance at which the curve reaches its inflection point in the thermal anomaly area. To improve the representativeness of this approach, additional numerical simulations were performed by varying the rock bridge length by a 10 cm increment between 0.3 m and 2.7 m. The results (Fig. 9a) clearly demonstrate that surface temperatures increase with increasing rock bridge length. However, it should be noted that beyond a rock bridge length of 1.7 m, increasing surface temperatures are no longer regular and tend to reach a limit value (Fig. 9a). We interpret this “saturation” as a geometric artifact since beyond 1.7 m, the rock bridge length becomes equal to or larger than the maximum thickness of the modeled geometry (1.8 m). The IP method provides the best results above a 0.7-m-long rock bridge; below this value, the FWHM method provides values closer to the real values (Fig. 9b). Nevertheless, the estimated lengths always remain smaller than the real lengths: on average, 0.11 m less with the IP and 0.17 m less with the FWHM (Fig. 9b).

**Figure 10:**
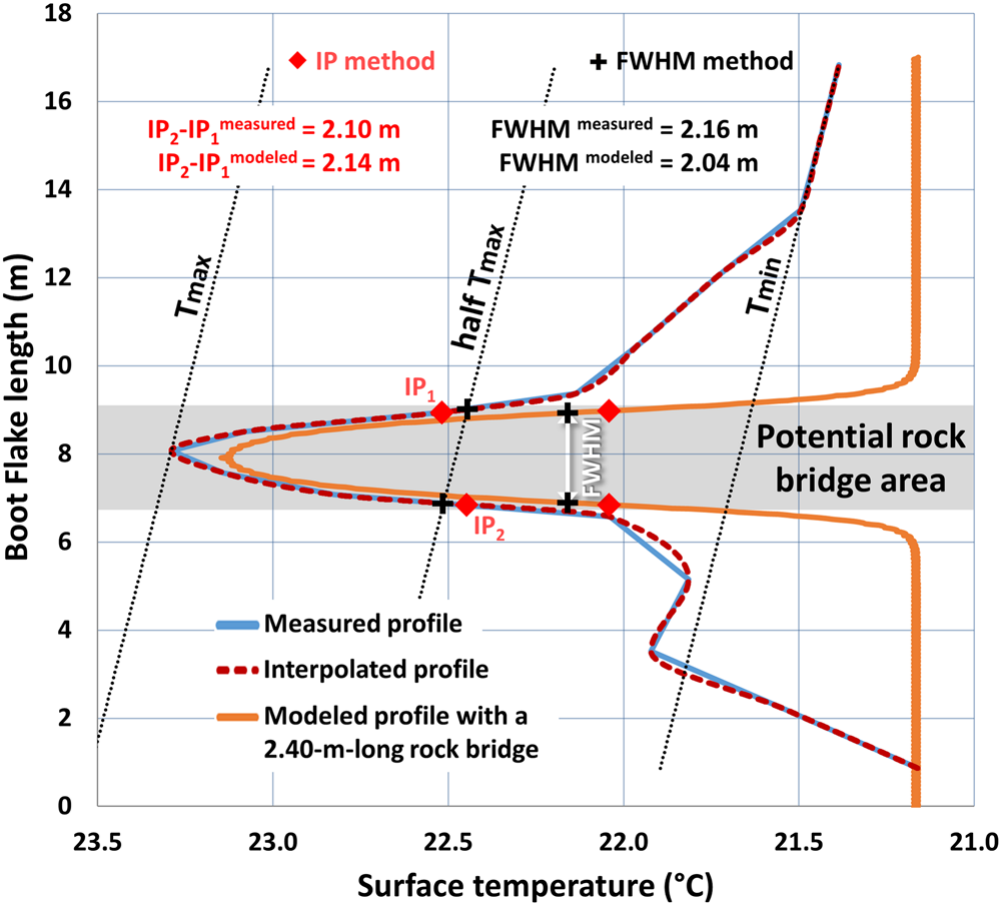
Comparison of the measured temperature profile for Boot Flake on 8 October 2015 at 17:45 PST with the modeled temperature profile with a 2.4-m-long rock bridge (results of the 12^th^ and final day of simulation at the exact same time). For the ease of comparison, all the temperatures of the modeled profile were reduced by 4.2 °C so that the minimum temperatures of measured and modeled profiles are identical. In addition, because the potential rock bridge is not exactly located in the center of Boot Flake, the position of the modeled profile has been moved downwards by 50 cm. To obtain a number of points similar to the modeled profile (i.e., one temperature value every 2 cm), the measured profile has been interpolated with a shape-preserving piecewise cubic interpolation (function PCHIP: Piecewise Cubic Hermite Interpolating Polynomial^123,124^ of MATLAB). The distances IP_2_-IP_1_^modeled^ and FWHM ^modeled^ (double white arrow) were determined using the interpolated profile.

By applying both slope-break profile methods to the full temperature profile of Boot Flake at 17:45 PST, we find a rock bridge length between 2.1 m (IP) and 2.16 m (FWHM) (Fig. 10). Given the calculated average errors for the IP and FWHM methods, these two estimates become, respectively, equal to 2.21 m and 2.33 m. Therefore, in accordance with the results of Fig. 8b, the new values are well below the 2.8 m initially measured (Fig. 5d) for the warm thermal anomaly detected at Boot Flake. They are, however, very close to those obtained (2.25 m and 2.21 m, respectively) with modeling a 2.4-m-long rock bridge (i.e., orange profile in Fig. 10). Thus, 2.4 m appears to be a very good approximation of the true length of the potential rock bridge of Boot Flake. Convincingly, the profile simulated with this rock bridge length reproduces almost exactly (2.0 °C instead of 2.1 °C) the maximum temperature difference measured along Boot Flake (Fig. 10). However, it should be noted that the shape of the modeled profiles does not reproduce the gradual decay of the surface temperatures observed between the rock bridge location and the flake edge (Fig. 10). The reasons for this difference and the consequences of this decrease in temperature on the stresses acting within the rear fracture are addressed in the Discussion section.

We take from these analyses that an overestimation of length of 0.4 m (2.8 m against 2.4 m) can be attributed to the IRT measurements. This suggests that for El Capitan Granite (the main rock type forming Boot and Texas flakes) a reduction factor of 1/7 should be applied to the thermal anomaly length to determine the true rock bridge length. Additionally, although we did not vary the rock bridge width in our thermal modeling, we assume that this reduction factor must similarly be applied to their width (i.e., 2.46 m against 2.11 m for Boot Flake). Thus, a reduction in surface area of 26.5% is necessary for determining more realistic rock bridge surface areas.

### Stability analysis

Knowing the dimensions of the rock bridges of Boot and Texas flakes, as revealed by the IRT/TLS analyses, offers an unprecedented opportunity to quantitatively analyze their stability. Here, we perform two types of analyses. The first uses fracture mechanics theory to compare the self-weight-induced stresses of the flakes to their ability to resist fracture at their respective rock bridge interfaces. The second analysis uses an empirical index of rock bridge attachment area to estimate the overall stability.

#### Fracture mechanics analysis of partially detached flakes

Brittle rock masses are subject to failure through propagation of fractures at their points of attachment (i.e., crack tips at rock bridges). As such, the use of fracture mechanics theory^109^ is well suited to analyzing flake stability. Here, we use linear elastic fracture mechanics to investigate the stability of Boot and Texas flakes. For simplicity, we assume that the only mechanism acting to cause instability is the self-weight of each flake’s rock mass, which drives gravitational forcing and subsequent outward rotation of the top of the flakes away from the cliff face (Fig. 7d,e). Thus, we envision the flake to be rotating outwards (inducing a tension at the top of the rock bridges). We idealize the somewhat complicated geometry of each of the flakes to coincide with geometrical fracture mechanics solutions available in the literature, and then compare the results (namely the stress intensity factor, *K*_*I*_ – a measure of the stress state at crack tips) to the measured fracture toughness values (*K*_*Ic*_) of the actual El Capitan rock mass (from laboratory testing). When *K*_*I*_ equals or exceeds *K*_*Ic*_, instability and rapid fracture (i.e., leading to rockfall) is expected. Exact solutions for Boot and Texas flake’s geometry are not available, however, a 3-D solution based on stress concentration factor methods^110^ ^(Eq. 25.7)^ captures the flakes fracture geometry fairly well (i.e., a disk-shaped attachment point of radius *b*, separating two parallel planes).

The fracture mechanics solution assumes that an outward moment (*M* = *mgΔ*) caused by the self-weight (*mg*) of the flake (0.50 MN for Boot; 3.37 MN for Texas) acts at a distance of *Δ* (0.21 m for Boot; 0.69 m for Texas) away from the point of attachment of the flake due to the separation of the base of the flake from the wall (Fig. 7d,e). The moment thus works to pry the partially-detached flake away from the parallel rock wall. Whereas the solution correctly mimics the geometry of Boot Flake, the analogy with Texas Flake is not quite correct since we know that Texas Flake is not cantilevered like Boot Flake (i.e., Texas Flake is partially supported at its base – Figs 1e and 7e). Still, this exercise is helpful for examining the role of rock bridges on the fracture geometry.

For Mode I (tensile) fracture, the stress intensity factor is given^110^ ^(Eq. 25.7)^ by:

1 $${K}_{I}=\frac{3}{2}\frac{M}{{b}^{2}\sqrt{\pi b}}\,\cos \,\theta $$

where *θ* is the orientation angle at which the stress is being evaluated along the edge of the rock bridge (maximum at *θ* = 0). For Boot Flake, the moment and radius length (*b*, equal to half the IRT-derived and corrected rock bridge length; i.e., 1/2 × 6/7 × 2.8 m) are, respectively, 0.11 MN·m and 1.2 m. For Texas Flake, these values are 2.33 MN·m and 0.8 m (*b* equal to 1/2 × 6/7 × 1.9 m). The resulting Mode I stress intensity factors are 0.06 MPa√m and 3.44 MPa√m, for Boot and Texas flakes, respectively.

The fracture toughness (*K*_*Ic*_ - measured as a critical stress intensity factor) of El Capitan Granite has recently been tested^111^ to be approximately 1.32 MPa√m. Thus, whereas *K*_*I*_ for Boot Flake is well below *K*_*Ic*_ (indicating stability), the stress intensity factor for Texas Flake exceeds *K*_*Ic*_ (indicating failure should have already occurred). We therefore believe the small rock bridge identified by the IRT analyses for Texas Flake is not the only point of attachment and explore this, as well as proximal features (the chockstones and the rock attachment area highlighted in Figs 1f and 7a), in more detail in the next section.

Fracture mechanics analysis also allows us to explore how failure might be reached at Boot Flake. If the rock bridge were to deteriorate and became smaller (decreasing *b* in Eq. 1), *K*_*I*_ would equal *K*_*Ic*_ when the rock bridge length (i.e., 2*b*) reached approximately 0.7 m (*b* = 0.34 m). Alternatively, critical fracture conditions could be reached if the loading condition changed (e.g., an increase in rotation moment by continued deflection (*Δ*) away from the wall or an increase in stresses beyond that by self-weight). This could take place, for example, by either thermal stress effects^12,13,112^ or the influence of water (or ice)^113–115^ pressure that could seasonally build up behind the flakes. Further, the fracture toughness that the stress intensity factors are evaluated against could be somewhat lower, given that the value used in our analyses is based on laboratory testing on relatively intact rock samples; the rock bridge interface is likely more highly weathered and would have a lower fracture toughness. Still, these analyses offer at least some insight into understanding the stability of the current geometry of these exfoliation sheets.

#### Evaluation of rock bridge attachment areas

Several post-rockfall event studies involving detailed mapping of rockfall scars^28,36,45^, characterization of discontinuity persistence^25,26,34,41,42,44,116^, and back analysis modeling^23,24,38,40,117–119^ have shown that rock bridge area (i.e., the presumed attachment area that holds a rock protrusion to the main cliff face) can vary between 0.2% and 45% of total scar surface, with an average value^28^ of about 22 ± 12%. To see where on this spectrum Boot Flake and Texas Flake may lie, we calculated the ratio between the maximum area of potential rock bridges and the total area of the exfoliation sheets.

The warm IRT anomaly observed on Boot Flake has a maximum length of 2.8 m (Fig. 5d) and a maximum surface area of 6.9 m^2^ (Fig. 7b). By applying the 26.5% IRT-based reduction factor (described previously) over the area of this anomaly, the effective maximum attachment area of the potential rock bridge is on the order of 5.1 m^2^. Given the total area of Boot Flake is equal to 75.5 m^2^ (Fig. 7b), the surface area of the rock bridge represents 6.8% of the total area. Thus, these results indicate that the rock bridge attachment area is about three times lower than the previously referenced^28^ average value, which ranks this value in the most “critical” part of the spectrum highlighted by the empirical data. However, the fracture mechanics analyses tell us otherwise, and for critical fracture conditions to be reached, the rock bridge area would need to reduce to 3.0 m² (for a 0.7 m long rock bridge and assuming a 4.3 m attachment width across the flake), representing only 4% of the total surface area of the flake.

We can perform similar analyses for Texas Flake. For the smaller IRT-identified rock bridge near the center of the flake, the effective (corrected) attachment area ([1–26.5%] × 2.0 m^2^ = 1.5 m^2^) is only 0.8% of the total area of Texas Flake (178.1 m^2^). This value is at the lower end of the 0.2% to 45% area of attachment spectrum identified in the literature and suggests (similar to the fracture mechanics results) that Texas Flake should be close to failure (or already detached completely). However, we also identified an additional rock attachment area from our IRT measurements along the base of the flake (Fig. 7a) with corrected attachment surface area of 13.1 m^2^ ([1–26.5%] × 17.8 m^2^). Aided by the IRT measurements, we deduce that the flake is attached here by approximately 7.3% of the total surface area – a result very similar to that calculated for Boot Flake (6.8%). Texas Flake further offers a unique opportunity to verify the model results, as the crack aperture behind the flake is sufficiently large to allow for direct inspection (indeed, the “Nose” climbing route travels between Texas Flake and the adjacent rock wall). Such inspection (Supplementary Fig. 2) reveals that the primary area of attachment is, in fact, at the base of the flake, represented by the warmest temperatures in Fig. 7c. Above this area, discrete, fractured rock sheets are in direct contact with the flake and the adjacent rock wall, providing a partial thermal link that appears as the purple and pink colors in Fig. 7c. By contrast, the uppermost chockstones highlighted in Fig. 1f, which are located 5.4 m above the 2.0 m² thermal anomaly (Supplementary Fig. 2), do not generate detectable thermal anomalies. A potential rock bridge or a compact wedged rock sheet that sits at the same location is therefore the likely source of the 2.0 m^2^ thermal anomaly in Fig. 7c. Thus, we assume that the dashed portion of Fig. 7e is actually intact rock across at least part of the width of the flake. The rock strength (at least in its unweathered form) and geometry of the two flakes therefore appear to be sufficient to resist outward gravitational forcing, even with only a ~7% rock bridge area connection to the cliff.

## Discussion

### IRT and thermal modeling comparison

Given the complexity of thermal imaging, and of course, from data collected in a natural setting, it is not surprising that discrepancies exist when comparing the IRT data to idealized thermal models. One significant difference between the results of the IRT monitoring and the 2-D thermal modeling is that the modeled temperatures are all higher than those measured (Figs 6b and 8b). This difference can come from the basic parameters used in the model to characterize the physical properties of the El Capitan Granite but also from the sources of external infrared radiation that affect the measurements of the calibrations parameters (more details are provided in Supplementary Appendix A). However, since the surface temperatures measured in the Boot Flake and Texas Flake areas have not been verified using thermoresistances, the IRT images are subject to a calibration bias that precludes identifying the source of the discrepancy between the observed and modeled temperatures. An additional substantial difference between the measured and modeled results is related to the shape of the modeled temperature profile which does not reproduce the “gradual” increase of the surface temperatures between the flake edge and the rock bridge location seen in the measured data (Fig. 10). On this flake’s portion, we observe a level of constant temperatures that mainly results from the constant thicknesses of the crack and flake applied to the model (Supplementary Appendix A). However, this is not the case in reality (Fig. 3b). In addition, it should be noted that this difference in behavior arises because the thermal conduction model is in 2-D, whereas 3-D effects obviously govern the actual thermal response of the flake and rock bridges. Finally, it is important to specify that the method used to measure the warm thermal anomaly size depends on several choices and manual procedures, including the determination of temperature thresholds, the texturing of IRT images on TLS data, and the drawing of 3-D polylines within the TLS data. In terms of uncertainty, a cumulative error of ±5% (Supplementary Appendix A) can be attributed to the estimate of the rock bridge surface at the end of this procedure.

### Potential of IRT monitoring for hazard assessment

Our monitoring of Boot and Texas flakes with IRT methods show that both flakes are always colder than the surrounding rock walls in the evening (Fig. 6). At the end of the day, exfoliation sheets with open cracks are characterized by colder thermal signatures (detection criterion) that result from air circulation that envelops and cools the detached parts (analogous to a heat transfer fin in engineering applications). Since the instruments used in our study can acquire IRT panoramas of 1,000-m-tall cliffs (Fig. 4), they could conceivably detect flakes over an entire cliff, thereby opening up a powerful new rock instability monitoring technique (assuming that partially detached flakes are more susceptible to rockfalls).

However, our study also revealed that cold thermal anomalies can exist for reasons other than partial detachment; these include areas with overhangs or with light-colored rocks. Consequently, classification of thermal anomalies must be conducted by superimposing^67,68^ the thermograms with digital photographs or by generating 3-D “thermo-photorealistic” models (Fig. 7). In addition, image rectification^120,121^ and topographic corrections must be applied to thermograms in order to account for the influence of lens distortion, incidence angle, spatial orientation, and emissivity for each pixel. Thus, utilizing our methodology for the purpose of examining an entire rock wall is not without substantial complications. That said, we have shown that coupled TLS-IRT data are very helpful for classifying topographic artifacts and for drawing 3-D maps of exfoliation sheets. Thus, we find the methodology worth exploring to determine the potential for estimating cliff-wide hazards. Even if this were to be found intractable (due to the investment needed in instrument and study area calibration), it might be used for long-term hazard assessment on a smaller scale. For example, capturing repeat surveys of the same areas of a rock cliff (e.g., repeating the data capture for Boot and Texas flakes) could possibly provide a path forward for IRT-based monitoring, in that the additional work required for calibration of the thermograms would not necessarily need to be performed in as rigorous detail each time data were collected. The repeat data might show trends in rock bridge area reduction (i.e., fracture growth). Coupled with fracture mechanics analyses like those we have presented, it may be possible to forecast when rock bridge area decreases such that the attachment length indicates fracture instability (i.e., when the stress intensity factor approaches the critical fracture toughness).

Recent studies^12,13^ have clearly shown the link between diurnal and seasonal temperature cycles and exfoliation flake movement. This is particularly true of thin, partially detached rock sheets that have the ability to deform. Boot and Texas flakes are such features, and being located on a south facing wall, may be particularly prone to show a thermal contrast between diurnal cycles. This, coupled with the fact that both flakes are significantly detached from the main rock cliff might suggest that they are ideal for identification and utilization with IRT methods. As we were able to verify that warmer areas did, in fact, coincide with rock attachment points, the corollary would hold that the method should be able to identify detached areas, even if the flakes are not as “open” as Boot and Texas flakes. Thus, it is plausible that less detached flakes might still be identifiable with IRT, even if their thermographic signal was not as strong. Obviously, additional calibration of the reflected signals would be needed. This might be particularly true were studies to be conducted on north-facing slopes where a smaller thermal differential (and stress) might be expected. Although our study did not incorporate thermal stresses into the analysis of stability, it is likely that a thermal signal could be contributing to the stresses acting on both Boot and Texas flakes. This, along with exploration of other possible stressors (e.g., the chockstone wedging mechanism^122^) could lead to higher stress intensity factors and possibly increased rock bridge deterioration. Thus, there are many avenues in need of further explanation; we expect the present study will assist with at least some of these.

## Conclusion

Quantifying rockfall hazards from steep cliffs continues to pose significant challenges, particularly due to our inability to measure the areal extent of rock bridges that fundamentally control the stability of partially detached rock masses. We explored the use of IRT technologies, in conjunction with TLS surveying, to remotely characterize the stability of two well-delimited granitic exfoliation sheets that form partially detached flakes on a nearly 1,000-m-tall vertical cliff. By examining the nocturnal cooling of the flakes, we found that both monitored flakes always remain colder than the surrounding rock walls. We interpret this colder thermal signature as a result of the air circulation that envelops and cools the detached parts from the cliff. This thermal signature could be used to improve the remote detection of partially detached exfoliation sheets in cliffs many hundreds of meters high, and from distances exceeding 1 km. Detailed analysis of our resulting thermograms for the flakes also revealed that the temperature patterns are not homogeneous – warmer thermal anomalies are visible on the surface of both monitored flakes. We interpret these local warmer areas as a sign of the presence of rock bridges that conduct heat from the cliff behind the exfoliation sheets. The detection of rock bridges from our IRT analysis is supported by the results of 2-D thermal modeling of an exfoliation sheet connected to the rock wall by a central rock bridge. By draping the IRT images on the TLS topographic data, we were able to measure the potential rock bridge surfaces and other areas of contact between the flakes and the adjacent wall, and thus evaluate flake-stability conditions. Based on the same geometric data, we supplemented this estimation with further analysis of fracture mechanics of both monitored exfoliation sheets. This study demonstrates that thermal imaging, combined with high-resolution 3-D topography, has significant potential for rockfall susceptibility and hazard analysis.

## Supplementary information

Supplementary Information

## Data Availability

The datasets generated during and/or analyzed during the current study are available from the corresponding author on reasonable request.
