# Supplementary material for: Detection of rock bridges by infrared thermal imaging and modeling
Source: Sci Rep. 2019 Sep 11;9:13138. doi: 10.1038/s41598-019-49336-1 (PMC6739350; doi:10.1038/s41598-019-49336-1)
Supplement: Supplementary file 1 — Supplementary Information [file 41598_2019_49336_MOESM1_ESM.pdf]

Detection of rock bridges by infrared thermal imaging and modeling

Antoine Guerin, Michel Jaboyedoff, Brian D. Collins, Marc-Henri Derron, Greg M. Stock, Battista Matasci, Martin Boesiger, Caroline Lefeuvre, and Yury Y. Podladchikov

Supplementary Appendix A: Data limitations

A.1 Limitations of the thermographic calibration protocol

The temperatures modeled at the Boot Flake surface are all higher than those measured between 17:45 and 21:40 PST with the IRT monitoring. For instance, the maximum temperature of the modeled profile at 17:45 PST is 27.5°C for a 2.4-m-long rock bridge whereas it was measured at 23.3°C (Fig. 5d). This difference can be explained by the choice of the basic physical properties (heat capacity at constant pressure and density; Table 2) proposed by COMSOL Multiphysics® software and used to characterize the El Capitan Granite in the 2-D thermal model. However, given the calibrated temperatures have not been verified, it is unclear whether the modeled values are overestimated or whether the measured values are underestimated.

For calibration, although relative humidity is not the most influential parameter, it should be noted that the very high values (>80%) recorded from 20:00 PST (Table 1) are most likely due to large herbs that shape El Capitan Meadow (Fig. 2a). They were damp at the end of the monitoring and it would have been preferable to position oneself at a non-vegetated location. However, we had no other choice because El Capitan Meadow is the only plain located as close to El Capitan, but far enough away from the trees that grow at the base of the El Capitan cliff. The evolution over time of both other calibration parameters (the reflected apparent temperature and ambient air temperature) seems consistent, both temperatures decline in a similar manner and the reflected apparent temperature is still lower than the ambient temperature (Table 1). However, as previously discussed, infrared radiation measured at the cliff foot is strongly influenced by incident ground radiation (Supplemental Fig. 1). As a consequence, the less dense warm air rising from the heated scree slopes all afternoon may have distort the reflected apparent temperature measurement.

A.2 Limitations of the 2-D thermal modeling

2-D thermal modeling very well reproduced the bell shape of measured temperature profiles. For a 2.4-m-long rock bridge, the maximum temperature difference along the modeled profile is 2.0°C (Fig. 10). This value is very close to the 2.1°C measured for Boot Flake at the same time (17:45 PST). In addition, although the modeled profile does not exactly reproduce the measured lengths with the IP (compare 2.14 m against 2.10 m) and FWHM methods (compare 2.04 m against 2.16 m), all the values are very similar (Fig. 10). However, the result of 2-D thermal modeling is not perfect because the "gradual" increase of the surface temperatures between the flake edge and the rock bridge location has not been reproduced (Fig. 10). The constant temperature modeled on the three-quarters of the flake probably results from the external forced convection flow applied to the flake-crack interface. In our case, the wind speed is the same on all the flake borders, whereas in reality, it must certainly be weaker and not homogeneous under the flake. In addition, the thicknesses of the crack and flake are not constant in reality (Fig. 3b). Thus, there are several intrinsic and extrinsic parameters of 2-D thermal modeling that greatly influence the shape and the final temperatures of the profiles.

A.3 Error sources influencing the warm thermal anomaly size measurement

The method we described here to estimate the warm thermal anomaly size depends on several choices and manual procedures. First, there is the determination of temperature thresholds to be applied to IRT images, then the selection of control points to drape IRT images over the TLS mesh and, finally, the 3-D polyline drawings that delineate the contours of the potential rock bridge areas. The first source of error is influenced by the value of the temperature threshold because it directly modifies the proportion of temperatures not taken into account on the thermograms (in black in Figs. 5c and 5g). In terms of uncertainty, an increase or decrease in the threshold

value of ± 0.05°C leads to a graphical error on the estimated length of ± 0.1m. The estimation of the size of the anomaly depends on the accuracy of the draping and contouring procedures, which are mainly influenced by the location of control points (they must be as far apart as possible). In the context of 3-D mapping of geological contacts, one study[124] investigated and compared the errors associated with these two procedures and found that the texturing stage appears to be the main source of error. However, the surfaces draped on El Capitan are much smaller, and the fact that a large part of the colder edges of Boot Flake and Texas Flake are black on the thermograms allows us to properly adjust the lateral limits of the flakes over the TLS mesh (Fig. 7). Finally, the delineation of 3-D polylines will always be different from one user to another and an uncertainty on the estimated surface of ± 5% can be attributed to the whole of this procedure.

Detection of rock bridges by infrared thermal imaging and modeling

Antoine Guerin, Michel Jaboyedoff, Brian D. Collins, Marc-Henri Derron, Greg M. Stock, Battista Matasci, Martin Boesiger, Caroline Lefeuvre, and Yury Y. Podladchikov

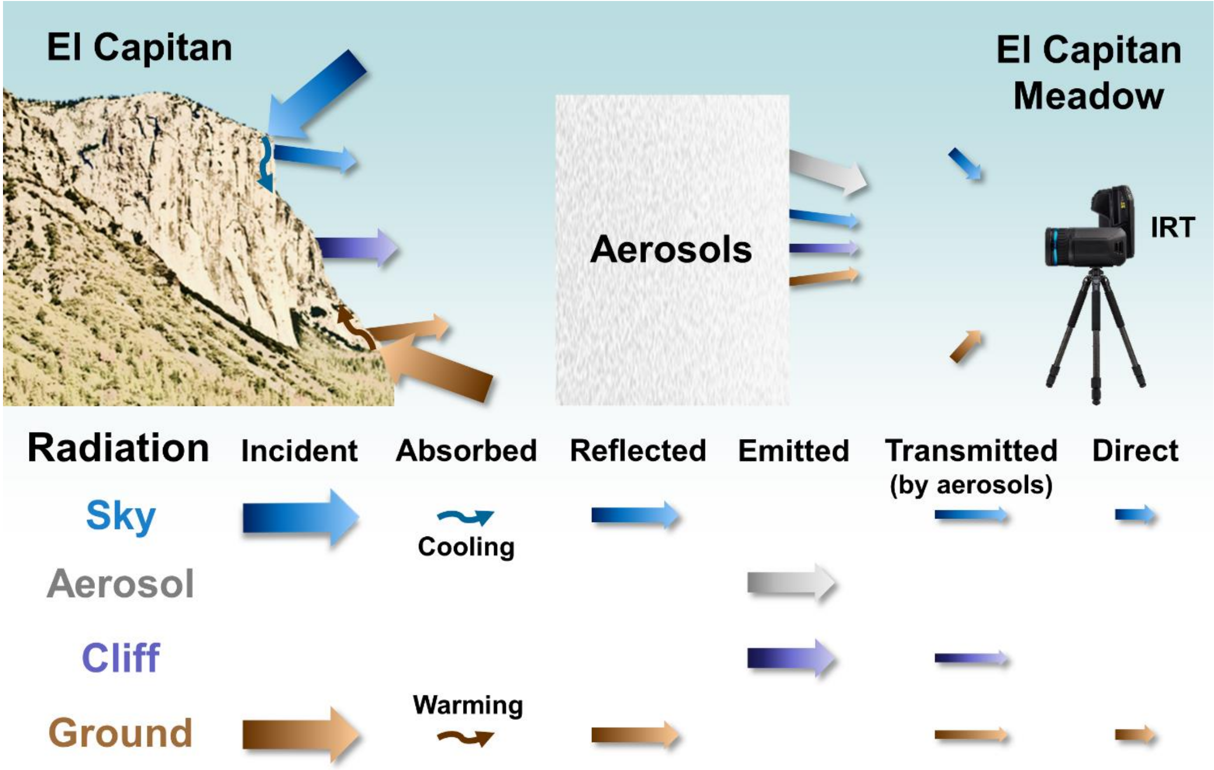

Supplementary Figure 1. Sketch of the thermographic measurement situation from El Capitan Meadow. The picture on the left shows the southwest face of El Capitan seen from Tunnel View (eastern gateway of Yosemite Valley). The picture on the right shows the FLIR T-660 infrared thermal camera mounted on a tripod.

Detection of rock bridges by infrared thermal imaging and modeling

Antoine Guerin, Michel Jaboyedoff, Brian D. Collins, Marc-Henri Derron, Greg M. Stock, Battista Matasci, Martin Boesiger, Caroline Lefeuvre, and Yury Y. Podladchikov

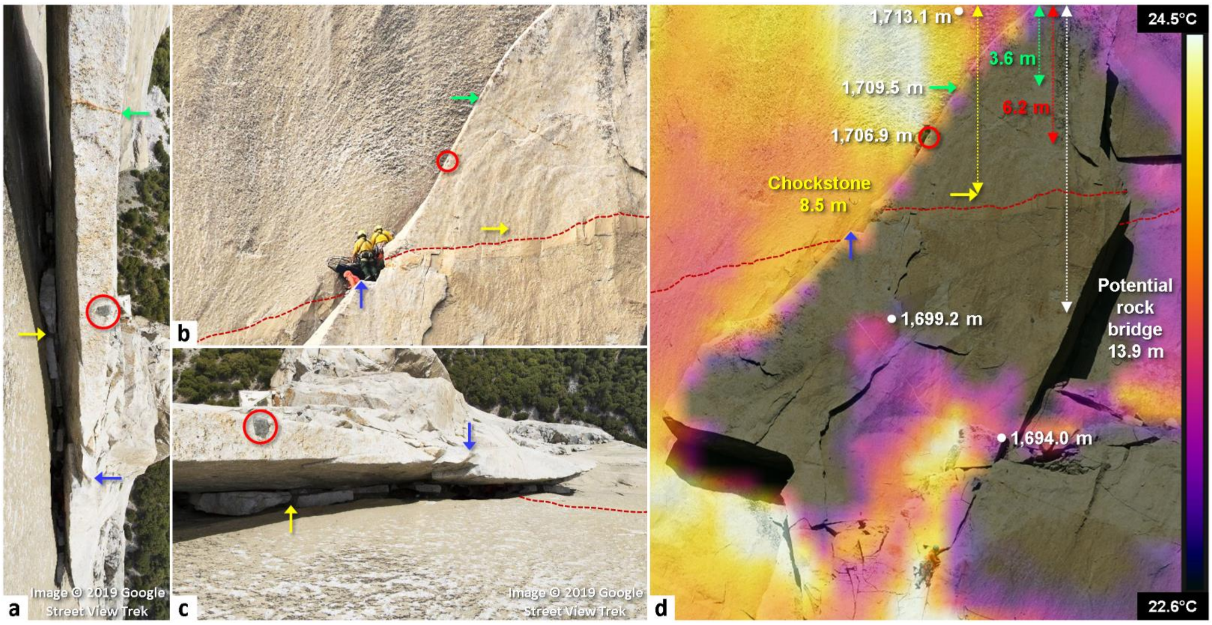

Supplementary Figure 2. Location of the uppermost chockstones of Texas Flake. (a) Profile view of the upper part of Texas Flake (west side); photographic credit: 2019 Google, El Capitan Street View Trek. The green and blue arrows and the red circle show the respective positions of the golden-colored vein, the small flat cornerstone and the diorite xenolith, used as benchmarks to determine the location of chockstones indicated by the yellow arrow. These four symbols show identical positions in Panels *b*, *c* and *d*. The golden-colored vein is 3.6 m below the top (1,713.1 m) of Texas Flake at 1,709.5 m and the diorite xenolith is 6.2 m below the top at 1706.9 m. This point of view shows that the chockstones are definitely below both of these features (8.5 m below the top of Texas Flake). **(b)** Front view of the upper west part of Texas Flake with rock climbers for scale; photographic credit: Thomas M. Evans (photograph reproduced under the CC BY 4.0 license). The red dashed line indicate the position of the upper limit of the aplite dike, used also as benchmarks to determine the location of the uppermost chockstones of Texas Flake. **(c)** High-angle view of the upper west side of Texas Flake; photographic credit: 2019 Google, El Capitan Street View Trek. This point of view shows that the aplite dike crosses in the same vicinity of the chockstones. **(d)** Front view of Texas Flake superimposed with the thermogram of Fig. 5g; photographic credit of the background image: Thomas M. Evans (photograph reproduced under the CC BY 4.0 license). The top of the potential rock bridge is 13.9 m below the top of Texas Flake at 1,699.2 m. This point of view shows that the location of the potential rock bridge is well below the aplite dike, and thus well below the chockstones.
